# BMP4 Moderates Glycolysis and Regulates Activation and Interferon-Gamma Production in CD4+ T Cells

**DOI:** 10.3389/fimmu.2021.702211

**Published:** 2021-08-03

**Authors:** Feng Huang, Lei Hu, Yuanmin Zhang, Xingmin Qu, Junji Xu

**Affiliations:** ^1^Department of Stomatology, Zhejiang Hospital, Hangzhou, China; ^2^Department of Prosthodontics, School of Stomatology, Capital Medical University, Beijing, China; ^3^Beijing Key Laboratory of Tooth Regeneration and Function Reconstruction, School of Stomatology, Capital Medical University, Beijing, China; ^4^Beijing Laboratory of Oral Health, Capital Medical University, Beijing, China; ^5^Immunology Research Center for Oral and Systemic Health, Beijing Friendship Hospital, Capital Medical University, Beijing, China; ^6^Department of Pediatric Dentistry, School of Stomatology, Capital Medical University, Beijing, China; ^7^Laboratory of Tissue Regeneration and Immunology and Department of Periodontics, School of Stomatology, Capital Medical University, Beijing, China

**Keywords:** BMP4, glycolysis, interferon-gamma (IFNg), CD4+ T activation, metabolism

## Abstract

BMP4 is a key growth factor well known in promoting bone regeneration and has been reported to be able to regulate T cell development in the thymus. Here, we showed that BMP4 downregulates the activation of naïve CD4+ T cells and the IFN-γ production of CD4+ T cells without increasing regulatory T cells. BMP4 could also moderate glycolysis of T cells and regulate Hif1α expression. Furthermore, BMP4 showed a suppressive function on the IFN-γ production of CD4+ T cells *in vivo*. These findings indicating a mechanism by which BMP-4 may regulate activation and IFN-γ production in CD4+ T cells *via* metabolism moderation and suggests that BMP4 may be a potential therapeutic supplement in autoinflammatory diseases.

## Introduction

Bone morphogenetic proteins (BMPs) are an important member of the transforming growth factor-β superfamily and play an important role in bone metabolism (1). They were initially identified as osteoinductive components in extracts derived from bone (2, 3), and now it has been discovered that different members of the BMP family have more extensive biological activities in a multitude of processes. These processes occur during embryonic development and adult homeostasis by regulating cellular lineage commitment, morphogenesis, differentiation, proliferation, and apoptosis of various types of cells throughout the body (4). Importantly, recent studies showed that some BMPs may regulate early thymocyte differentiation (5) and inhibit early T cell development (6), and also may inhibit T cell activation and differentiation (7). However, the mechanism behind this pathway is still unknown.

Aerobic glycolysis, which is the first of three stages that make up aerobic cellular respiration, is a hallmark property of T cells where the cells rely on glycolytic pathways to meet their high demand for energy and for the proliferation of Interferon-gamma (IFN-γ) cytokine production (8). Glycolysis is a common cellular respiratory pathway that takes place in the cytoplasm. It begins with a molecule of glucose and is progressively broken down *via* various enzymes with final yields of 2 ATPs, 2 NADH2, and 2 pyruvic acid molecules. There are 10 enzymes that act as catalysts in different steps of the process of glycolysis, which may potentially be regulated by Hif1a and c-Myc. Studies have identified that BMPs regulate hypoxia-inducible factor 1 alpha (Hif1α) (9) and c-Myc (10), indicating that BMPs may regulate T cell proliferation and cytokine production *via* metabolism moderation.

BMP4 is a member of the BMP family and is of special interest due to its well-studied osteoinductive properties. Here, we showed the effects of BMP4 on the activation and differentiation of CD4+ T cells, so as to analyze the possible mechanisms of how BMP4 affects T cells metabolism. Moreover, administration of BMP4 *in vivo* can also suppress the IFN-γ production in CD4+ T cells in lymph nodes. Our findings reveal the possible mechanism of the regulatory function of BMP4 on CD4+ T cells and may have implications for the development of immunotherapy for clinical use.

## Materials and Methods

### Mice

C57BL/6 mice were obtained from Vital River, CD45.1 mice and OTII mice were obtained from Cyagen and bred in our facility under specific-pathogen-free conditions. All mice used for experiments were aged 6-12 weeks. All animal studies were performed according to guidelines for the use and care of live animals and were approved by the Animal Care and Use Committees of Capital Medical University.

For *In Vivo* experiment, totally 18 CD45.1 transgenic C57BL/6 mice (male, 8 weeks old) were separated into 3 groups (n=6) and served as recipients, 3 OTII (male, 8 weeks old) mice were served as donors of CD4+ T cells. OVA peptide (pOVA 323-339, Sangon Biotech) was injected in footpads 10 μg/mouse. BMP4 (PeproTech) was administrated in 25 ng/µL for footpad injection in 10 µL. BMP4 neutralizing antibody (R&D system) was used in 10mg/kg for i.p.

### T Cell Culture

Naïve mouse (CD4+CD62L+CD44−) T cells were purified by magnetic cell sorting (Miltenyi Biotec) and stimulated with indicated concentration of plate-bound anti-CD3 and anti-CD28, in complete DMEM with 10% FBS, 2mM L-glut, 100 units/mL penicillin G, 100 µg/mL streptomycin, 1 mM sodium pyruvate, 10mM HEPES and Non-Essential Amino Acid (NEAA), cultured at 37°C for the indicated durations. BMP4 (PeproTech) was used in 10ng/ml for cell culture.

### Real-Time PCR

Total RNA was derived from cells using RNAsimple Kit (Tiangen) following the manufacturer’s instructions, and complementary DNA (cDNA) was synthesized from 100 ng of total RNA using the Reverse Transcription Kit (ThermoFisher). Quantitative real-time PCR was analyzed using the δδ-Ct method. Primer were used as follows: *Hprt* (Mm03024075_m1; Invitrogen), *Il2ra* (Mm01340213_m1; Invitrogen), *Cd69* (Mm01183378_m1; Invitrogen), *Cd44* (Mm01277165_m1; Invitrogen), *Ifng* (Mm01168134_m1; Invitrogen), *Il4* (Mm00445259_m1; Invitrogen), *Foxp3* (Mm00475162_m1; Invitrogen), *Hk1* (Mm01145244_m1; Invitrogen), *Hk2* (Mm00443385_m1; Invitrogen), *Hk3* (Mm01341942_m1; Invitrogen), *Pfkl* (Mm00435597_m1; Invitrogen), *Myc* (Mm00487804_m1; Invitrogen), *Hif1a* (Mm00468869_m1; Invitrogen).

### Flow Cytometry Analysis

Antibodies for FACS were purchased from ThermoFisher eBioscience: Anti-CD4 mAbs, clone: RM4-5; Anti-CD25 mAbs, clone: PC61.5; Anti-CD69 mAbs, clone: H1.2F3; Anti-CD44 mAbs, clone: IM7; Anti-IL-4 mAbs, clone: 11B11; Anti-IFN-g mAbs, clone: XMG1.2; Anti-FoxP3 mAbs, clone: FJK-16S. Intranuclear staining was carried out using the Fixation/Permeabilization buffer solution (eBioscience) according to manufacturer’s instruction. For intracellular cytokine staining, cells were stimulated with PMA (50 ng/ml), Ionomycin (250 ng/ml) and Golgi-Plug (1:1000 dilution, BD PharMingen) at 37°C for 3 hr, followed by fixation with the Fixation/Permeabilization buffer solution (BD Biosciences) according to manufacturer’s instruction. Stained cells were analyzed on a LSRFortessa (BD Biosciences) and data was analyzed with FlowJo software.

### Metabolic Analysis

Extracellular acidification rate (ECAR) and oxygen consumption rate (OCR) of CD4+ T cells were measured using a Seahorse XFe96 Analyzers. T cells were cultured with or without BMP4 (10ng/ml, PeproTech) for 24 hours, and then were prepared in XF RPMI medium supplemented with 10 mM glucose, 1 mM pyruvate and 2 mM L-glutamine, added 2 × 10^5^ T cells into each well of Seahorse XF96 cell culture microplates (Coated with 22.4 μg/ml Corning^®^ Cell-Tak Cell and Tissue Adhesive), spined down and preincubated at 37°C for around 30 min in the absence of CO^2^, then ran the program of the Seahorse XF Cell Mito Stress Test.

### Statistical Analysis

Comparison between two different groups was done by unpaired two-tailed Student’s t test; Comparisons between more than two groups were done by one-way ANOVA (with Tukey’s multiple-comparisons post-tests). All P values less than 0.05 were considered significant. Statistical analysis was done with GraphPad Prism 7.

## Results

### BMP4 Downregulates the Activation of Naïve CD4+ T Cells

**Figure 1:**
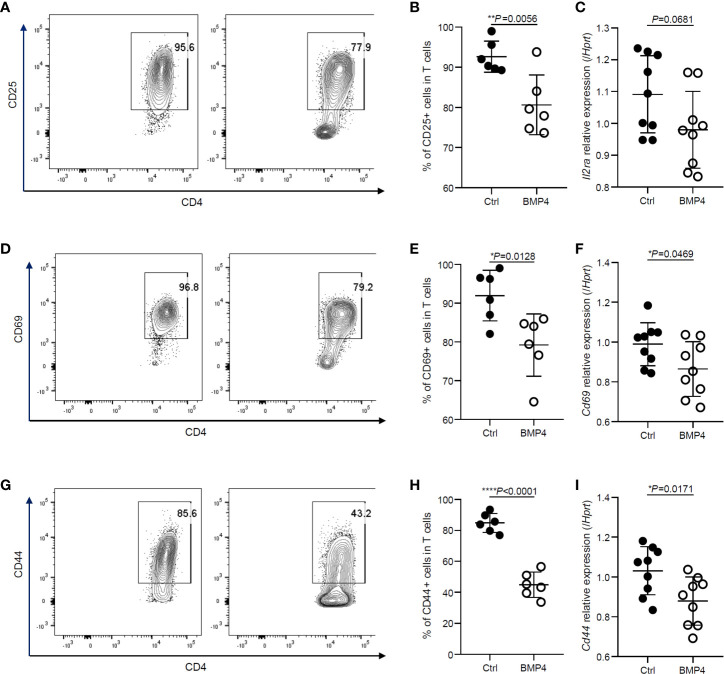
BMP4 downregulates the activation of naïve CD4+ T cells. **(A)** The expression of CD25 on T cells tested by flow cytometer 24 hours after TCR activation with or without BMP4 treatment. **(B)** The frequency of CD25+ T cells. **(C)** The gene expression of *Il2ra* checked by Quantitative real-time PCR. **(D)** The expression of CD69 on T cells. **(E)** The frequency of CD69+ T cells. **(F)** The gene expression of *Cd69* checked by Quantitative real-time PCR. **(G)** The expression of CD44 on T cells. **(H)** The frequency of CD44+ T cells. **(I)** The gene expression of *Cd44* checked by Quantitative real-time PCR.

We first examined the effects of BMP4 on the activation of naïve CD4+ T cells. CD25, CD69, and CD44 are commonly used activation markers of the CD4+ T cells. After T cell receptor (TCR) stimulation for 24 hours, usually, more than 90% of the naïve T cells become CD25+ (**Figures 1A, B**) when analyzed by flow cytometry. BMP4 treatment along with TCR activation could downregulate the expression of CD25 on T cells (**Figures 1A, B**). The gene expression of *Il2ra* was also checked by PCR, although it was not found to be significant and it showed a trend of decrease in *Il2ra* after BMP4 treatment (**Figure 1C**). T cells also have high levels of CD69 and CD44 expression 24 hours after TCR stimulation (**Figures 1D, G**), and the frequency of CD69+ or CD44+ T cells declined while BMP4 was administered in the culture system (**Figures 1E, H**). We also double-checked the gene expression of *Cd69* and *Cd44* (**Figures 3F, I**), which also significantly decreased after BMP4 treatment. Taken together, our data showed that BMP4 could downregulate the activation of naïve CD4+ T cells.

### BMP4 Downregulates the IFN-γ Production of CD4+ T Cells Without Increasing Tregs

**Figure 2:**
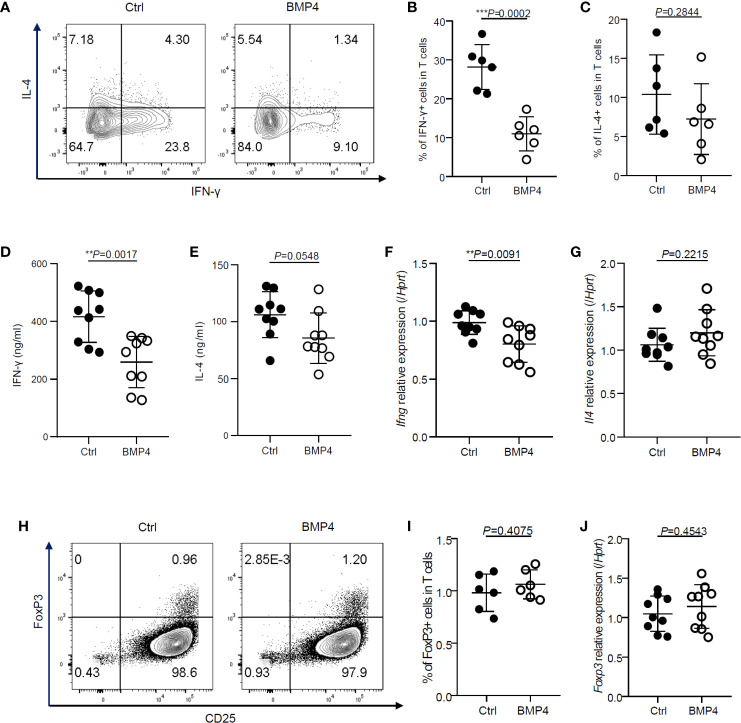
BMP4 downregulates the IFN-γ production of CD4+ T cells without increasing Tregs. **(A)** The expression of IFN-γ and IL-4 on T cells were tested by flow cytometer 3 days after TCR activation with or without BMP4 treatment. **(B)** The frequency of IFN-γ+ T cells. **(C)** The frequency of IL-4+ T cells. **(D)** The concentration of IFN-γ+ in the supernatant of T cells culture medium 3 days after TCR activation with or without BMP4 treatment. **(E)** The concentration of IFN-γ+ in the supernatant of T cells culture medium. **(F)** The gene expression of *Ifng* checked by Quantitative real-time PCR. **(G)** The gene expression of *Il4*. **(H)** The expression of FoxP3 on T cells tested by flow cytometer. **(I)** The frequency of FoxP3+ T cells. **(J)** The gene expression of *Foxp3*.

We next examined the administration of BMP4 and its effects upon IFN-γ production of CD4+ T cells. After activation together with treatment of BMP4 for 3 days, the production of IFN-γ was decreased. Our flow cytometry data showed that the ratio of IFN-γ producing CD4+ T cells significantly decreased after BMP4 administration compared with blank control (**Figures 2A, B**). The BMP4 treated CD4+ T cells also showed a decreasing trend in the ratio of IL-4 production (**Figures 2A, C**). The ELISA test for supernatants (**Figures 2D, E**) and PCR for *Ifng* and *Il4* gene expression (**Figures 2F, G**) also confirmed that BMP4 could downregulate the IFN-γ production of CD4+ T cells.

It is well known that TGF-β can induce T cells to differentiate into regulatory T cells (Tregs) (11), which can dramatically suppress the T cells activation and IFN-γ production (12). Since BMP4 is a member of the TGF-β superfamily, we hypothesize that the BMP4 may also induce Tregs. Surprisingly, the expression FoxP3, which is a crucial intracellular marker and also a key developmental and functional factor for CD4+CD25+ Tregs, did not show any significant change in CD4+ T cells after BMP4 treatment when compared to blank control, as analyzed by flow cytometry (**Figures 2H, I**) and PCR (**Figure 2J**). These data demonstrate that BMP4 downregulates the activation and IFN-γ production of CD4+ T cells without increasing Tregs.

### BMP4 Moderates Glycolysis of T Cells After Activation

**Figure 3:**
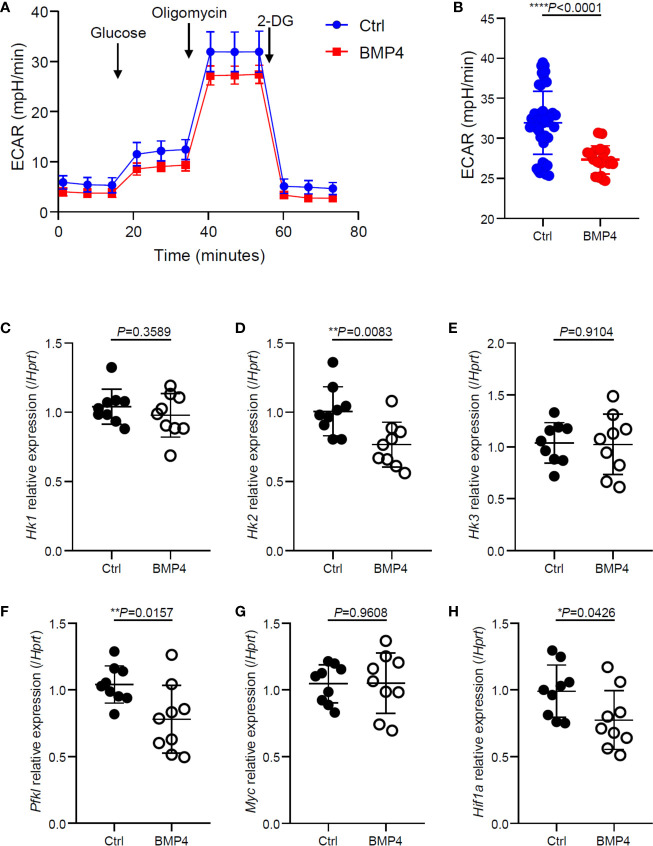
BMP4 moderates glycolysis of T cells after activation. **(A, B)** Extracellular acidification rate of T cells 24 hours after TCR activation with or without BMP4 treatment. **(C–H)** The gene expression of *Hk1, Hk2, Hk3, Pfkl, Myc* and *Hif1a* checked by Quantitative real-time PCR.

The activation and cytokine production of T cells relies upon glycolytic pathways to meet their high energy demand. To further investigate the mechanism of regulatory effects on T cells by BMP4, we investigated the glycolysis of T cells that had been activated for 24 hours. Glycolysis stress test measuring extracellular acidification rate (ECAR) is completed by injecting glucose to glucose-starved cells, followed by oligomycin and 2-deoxyglucose injections showing significantly lower glycolytic capacity in BMP4 treated T cells (24 hours) compared to blank control T cells (**Figures 3A, B**). PCR data showed that BMP4 may decrease the gene expression of hexokinases (*Hk2*) and pyruvate dehydrogenase kinase, isozyme 1 (*Pdk1*) in CD4+ T cells (**Figures 3C–F**), which are two important enzymes for glycolysis. Moreover, BMP4 also decreased the gene expression of *Hif1a*, which is one of the most important moderators of glycolysis, bother than c-Myc (**Figures 3G, H**). Taken together, our data suggested that BMP4 may moderate glycolysis of T cells *via* regulating Hif1α.

### BMP4 Suppresses the IFN-γ Production in CD4+ T Cells *In Vivo*

**Figure 4:**
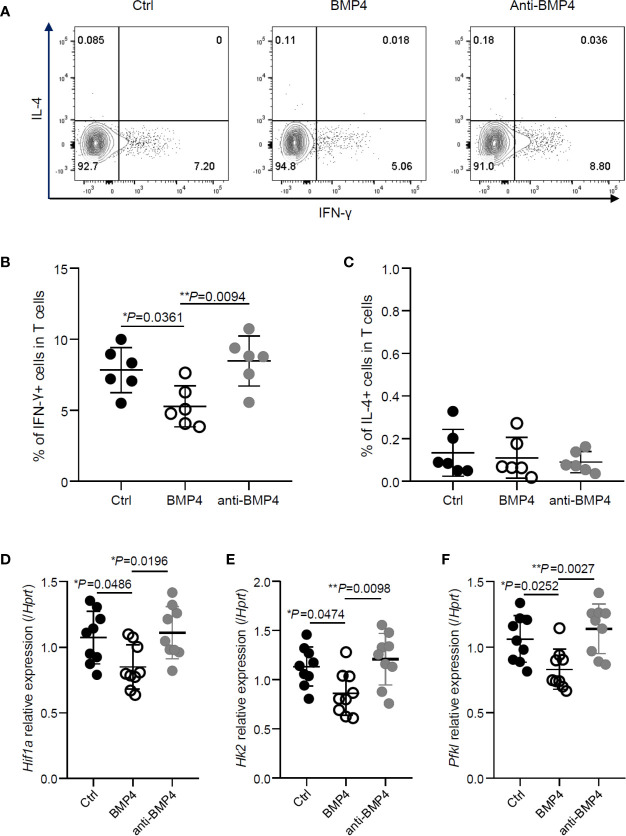
BMP4 suppresses the IFN-γ production in CD4+ T cells *in vivo*. **(A)** OT-II CD4+ T cells were adoptively transferred to CD45.1 transgenic C57BL/6 mice and injected OVA peptides into the footpads. The expression of IFN-γ and IL-4 on T cells from the draining lymph nodes of footpads with or without administration of BMP4 or anti-BMP4 antibody treatment. **(B)** The frequency of IFN-γ+ T cells. **(C)** The frequency of IL-4+ T cells. **(D–F)** The gene expression of *Hif1a, Hk2, Pfkl* checked by Quantitative real-time PCR.

To investigate the effects of BMP4 on CD4+ T cells *in vivo*, we adoptively transferred OT-II CD4+ T cells to CD45.1 transgenic C57BL/6 mice and injected OVA peptides into the footpads. Administration of BMP4 treatment significantly reduced the IFN-γ production in CD4+ T cells from the draining lymph nodes of footpads, while anti-BMP4 antibody injection induced the IFN-γ production to slightly increased (**Figures 4A, B**). The IL-4 production was also checked, however no significant change could be observed (**Figures 4A, C**). To further confirm the effects of BMP4 on glycolysis, total CD4+ T cells were isolated from draining lymph nodes by magnetic cell isolation, and PCR showed that the metabolism-related genes (*Hif1a*, *Hk2*, and *Pdk1*) in the T cells from BMP4 treated mice have lower expression than both blank control and anti-BMP4 treated mice (**Figures 4D–F**). Together, these data showed that BMP4 could suppress the IFN-γ production in CD4+ T cells *in vivo*.

## Discussion

BMP4 is a key growth factor well known to promote bone regeneration, and was reported to regulate T cell development in the thymus (5, 6). However, it was not clear whether BMP4 moderates the peripheral T cells or how the possible mechanism proceeds. In this study, we revealed that BMP4 downregulates the activation of naïve CD4+ T cells and the IFN-γ production of CD4+ T cells. It was unclear how BMP4 achieves the regulatory function since Tregs may not be induced by BMP4. We showed here that BMP4 moderated glycolysis of T cells *via* regulating Hif1α expression. Furthermore, BMP4 could suppress the IFN-γ production in CD4+ T cells *in vivo*. Therefore, our *in vitro* and *in vivo* results demonstrate that BMP4 regulates activation and IFN-γ production in CD4+ T cells *via* metabolism moderation.

It is already known that glycolysis is required for IFN-γ cytokine production in T cells (8), and that it promotes T helper 1 cell differentiation through an epigenetic mechanism (13). T cells also require pyruvate, which is majoritively produced during aerobic glycolysis, to initial oxidative phosphorylation and support the T cells activation (8). Moreover, activated T cells need the energy produced by glycolysis to fuel proliferation (8). Our data showed that BMP4 could moderate glycolysis of CD4+ T cells and significantly downregulate the activation of naïve CD4+ T cells. The IFN-γ production of CD4+ T cells was also dramatically downregulated by BMP4 without increasing Tregs. Together, these data suggested that BMP4 downregulates the activation and production of IFN-γ by moderating the glycolysis of CD4+ T cells.

Hypoxia-inducible factor 1-alpha (Hif1α) is a transcription factor that plays an important role in the cellular response to systemic oxygen levels in mammals (14). Metabolomics analysis showed that Hif1α could induce glycolysis metabolism and pentose phosphate pathway (15) by controlling the expression of members of the glycolytic cascade, including hexokinase II (HK2) (16, 17) and pyruvate dehydrogenase kinase, isozyme 1 (PDK1) (18). Hexokinases phosphorylate glucose to produce glucose-6-phosphate, which is the first step in most glucose metabolism pathways, and the PDK1 controls the switch of glucose metabolism from aerobic oxidation to glycolysis. Our data showed that BMP4 could downregulate the glycolysis and the expression of *Hif1a*, *Hk2*, and *Pdk1*, indicating that BMP4 may moderate glycolysis of CD4+ T cells by controlling Hif1α and glycolysis related enzymes. Further studies were needed to address the mechanisms of how BMP4 regulates the expression and function of Hif1α.

It is well known that the TGF-β superfamily has powerful regulatory functions on T cells. Different TGFβ superfamily members may have overlapping functions, but they can also be highly specific or even oppose the functions of each other (19). TGF-β can dramatically induce T cells to differentiate into Tregs (11) and suppress the activation and proliferation of T cells (20). BMPs also play a role in the differentiation and proliferation of mature T cells in the periphery (19), however, the mechanisms still need to be elucidated. Here, we presented that BMP4 regulates activation and IFN-γ production of CD4+ T cells, we also showed BMP4 may moderating glycolysis and Hif1α expression. Our results might help us to understand the molecular mechanisms of BMPs on T cells and identify the new therapeutic targets for autoimmune diseases and tumors.

## Data Availability Statement

The raw data supporting the conclusions of this article will be made available by the authors, without undue reservation.

## Ethics Statement

The animal study was reviewed and approved by the Animal Care and Use Committees of Capital Medical University.

## Author Contributions

FH and LH designed and performed most of the experiments, analyzed and interpreted the data. YZ and XQ performed experiments. JX conceived and supervised the whole study, designed experiments, and wrote the manuscript. All authors contributed to the article and approved the submitted version.

## Funding

This study was supported by grants from the National Science Foundation of China (81300896 to JX, 82001067 to LH); Beijing Municipal Natural Science Foundation (7142069 to JX); and Beijing NOVA program (2015B062 to JX).

## Conflict of Interest

The authors declare that the research was conducted in the absence of any commercial or financial relationships that could be construed as a potential conflict of interest.

## Publisher’s Note

All claims expressed in this article are solely those of the authors and do not necessarily represent those of their affiliated organizations, or those of the publisher, the editors and the reviewers. Any product that may be evaluated in this article, or claim that may be made by its manufacturer, is not guaranteed or endorsed by the publisher.

## References

[1] Chen D Zhao M Mundy GR. Bone Morphogenetic Proteins. Growth Factors (2004) 22:233–41. 10.1080/08977190412331279890 15621726

[2] Urist MR. Bone: Formation by Autoinduction. Science (1965) 150:893–9. 10.1126/science.150.3698.893 5319761

[3] Wozney JM Rosen V Celeste AJ Mitsock LM Whitters MJ Kriz RW. Novel Regulators of Bone Formation: Molecular Clones and Activities. Science (1988) 242:1528–34. 10.1126/science.3201241 3201241

[4] Katagiri T Watabe T. Bone Morphogenetic Proteins. Cold Spring Harb Perspect Biol (2016) 8. 10.1101/cshperspect.a021899 PMC4888821 27252362

[5] Cejalvo T Sacedon R Hernandez-Lopez C Diez B Gutierrez-Frias C Valencia J. Bone Morphogenetic Protein-2/4 Signalling Pathway Components are Expressed in the Human Thymus and Inhibit Early T-Cell Development. Immunology (2007) 121:94–104. 10.1111/j.1365-2567.2007.02541.x 17425602 PMC2265915

[6] Hager-Theodorides AL Outram SV Shah DK Sacedon R Shrimpton RE Vicente A. Bone Morphogenetic Protein 2/4 Signaling Regulates Early Thymocyte Differentiation. J Immunol (2002) 169:5496–504. 10.4049/jimmunol.169.10.5496 12421925

[7] Yoshioka Y Ono M Osaki M Konishi I Sakaguchi S. Differential Effects of Inhibition of Bone Morphogenic Protein (BMP) Signalling on T-Cell Activation and Differentiation. Eur J Immunol (2012) 42:749–59. 10.1002/eji.201141702 22144105

[8] Chang CH Curtis JD Maggi LB Jr. Faubert B Villarino AV O’Sullivan D. Posttranscriptional Control of T Cell Effector Function by Aerobic Glycolysis. Cell (2013) 153:1239–51. 10.1016/j.cell.2013.05.016 PMC3804311 23746840

[9] Lee SY Abel ED Long F. Glucose Metabolism Induced by Bmp Signaling Is Essential for Murine Skeletal Development. Nat Commun (2018) 9:4831. 10.1038/s41467-018-07316-5 30446646 PMC6240091

[10] Liu L Wei X Huang R Ling J Wu L Xiao Y. Effect of Bone Morphogenetic Protein-4 on the Expression of Sox2, Oct-4, and C-Myc in Human Periodontal Ligament Cells During Long-Term Culture. Stem Cells Dev (2013) 22:1670–7. 10.1089/scd.2012.0548 23311397

[11] Chen W Jin W Hardegen N Lei KJ Li L Marinos N. Conversion of Peripheral CD4+CD25- Naive T Cells to CD4+CD25+ Regulatory T Cells by TGF-Beta Induction of Transcription Factor Foxp3. J Exp Med (2003) 198:1875–86. 10.1084/jem.20030152 PMC2194145 14676299

[12] Chen W Wahl SM. TGF-Beta: The Missing Link in CD4+CD25+ Regulatory T Cell-Mediated Immunosuppression. Cytokine Growth Factor Rev (2003) 14:85–9. 10.1016/S1359-6101(03)00003-0 12651220

[13] Peng M Yin N Chhangawala S Xu K Leslie CS Li MO. Aerobic Glycolysis Promotes T Helper 1 Cell Differentiation Through an Epigenetic Mechanism. Science (2016) 354:481–4. 10.1126/science.aaf6284 PMC5539971 27708054

[14] Semenza GL. Regulation of Mammalian O2 Homeostasis by Hypoxia-Inducible Factor 1. Annu Rev Cell Dev Biol (1999) 15:551–78. 10.1146/annurev.cellbio.15.1.551 10611972

[15] Wang T Liu H Lian G Zhang SY Wang X Jiang C. HIF1alpha-Induced Glycolysis Metabolism Is Essential to the Activation of Inflammatory Macrophages. Mediators Inflamm (2017) 2017:9029327. 10.1155/2017/9029327 29386753 PMC5745720

[16] Semenza GL. HIF-1 Mediates Metabolic Responses to Intratumoral Hypoxia and Oncogenic Mutations. J Clin Invest (2013) 123:3664–71. 10.1172/JCI67230 PMC3754249 23999440

[17] Mergenthaler P Kahl A Kamitz A van Laak V Stohlmann K Thomsen S. (HKII) and Phosphoprotein Enriched in Astrocytes (PEA15) Form a Molecular Switch Governing Cellular Fate Depending on the Metabolic State. Proc Natl Acad Sci USA (2012) 109:1518–23. 10.1073/pnas.1108225109 PMC3277118 22233811

[18] Semba H Takeda N Isagawa T Sugiura Y Honda K Wake M. HIF-1alpha-PDK1 Axis-Induced Active Glycolysis Plays an Essential Role in Macrophage Migratory Capacity. Nat Commun (2016) 7:11635. 10.1038/ncomms11635 27189088 PMC4873978

[19] Chen W Ten Dijke P. Immunoregulation by Members of the TGFbeta Superfamily. Nat Rev Immunol (2016) 16:723–40. 10.1038/nri.2016.112 27885276

[20] Tu E Chia CPZ Chen W Zhang D Park SA Jin W. T Cell Receptor-Regulated TGF-beta Type I Receptor Expression Determines T Cell Quiescence and Activation. Immunity (2018) 48:745–759 e6. 10.1016/j.immuni.2018.03.025 29669252 PMC5911925

